# Burden and Anxiety in Family Caregivers in the Hospital That Debut in Caregiving

**DOI:** 10.3390/ijerph16203977

**Published:** 2019-10-18

**Authors:** Margarita Pérez-Cruz, Laura Parra-Anguita, Catalina López-Martínez, Sara Moreno-Cámara, Rafael del-Pino-Casado

**Affiliations:** 1 University Hospital “Dr. Sagaz”, Jaén, 23071 Jaén, Spain; mperezc23@gmail.com; 2 Department of Nursing, School of Health Sciences, University of Jaén, 23071 Jaén, Spain; cmartine@ujaen.es (C.L.-M.); smcamara@ujaen.es (S.M.-C.); rdelpino@ujaen.es (R.d.-P.-C.)

**Keywords:** hospital, caregivers, subjective burden, anxiety

## Abstract

This cross-sectional study aims to determine the level of subjective burden and anxiety of caregivers of dependent older relatives that start providing care in the hospital and to analyse the relationship between objective burden, subjective burden and anxiety in these caregivers. Seventy-two caregivers of dependent older relatives were recruited in a medium–long stay hospital. Sociodemographic variables, number of basic activities of daily living (ADLs) attended, hours of surveillance, burden, and anxiety were collected from caregivers. A trajectory analysis was used to analyse the relationship between variables. Of the caregivers, 36.1% had subjective burden and 14.9% had anxiety. Subjective burden was positively associated with the number of basic ADLs attended, the hours of surveillance, and the cognitive impairment of the care recipient. Anxiety was also positively associated with subjective burden. Subjective burden mediated the effects of the number of basic ADLs attended, hours of surveillance and the cognitive impairment of the care recipient on anxiety. The levels of subjective burden and anxiety in caregivers debuting in hospital care are elevated, showing the need for these caregivers to be cared for. Subjective burden is a possible risk factor for anxiety, independent of the objective burden; it may buffer the effects of objective burden on anxiety.

## 1. Introduction

The aging of the population in recent decades has led to an important increase in situations of dependence and the need for long-term care. This care tends to be predominantly carried out by family [1]. Within the nuclear family, it is traditionally the woman that takes on the majority of this care [2].

This increase in the demand for long-term care has resulted in the creation of hospitals or units for medium–long stay or convalescence. These hospitals and units are very useful for their effectiveness in providing care for the dependent patient that requires hospitalization, as in these units, they provide care focused on patient adaptation to the disability, or the involvement and teaching of family members in the care of dependent older persons [3].

### 1.1. The Caregivers in the Hospital

Immersion in the hospital structure and life causes an important change in a person’s usual role, as they are required to adapt to an environment which is usually unknown. This new situation affects both the life of the person admitted to hospital and the person providing care [4,5]. In the hospital environment, it also tends to be a woman who remains with the patient during the hospital stay, taking on the responsibility of care without economic remuneration for that—she is the hospital family caregiver [6,7]. Family caregivers provide emotional and instrumental support to the relatives, help maintain their safety, and care for their basic activities of daily living [6,7].

Studies show that family caregivers in the hospital spend many hours in the hospital and the intensity of the care they provide is high [8]. This figure is also invisible to the healthcare system, as they are not recognized as providers of care during hospital admission, let alone considered as users of the system [4,9,10]. They do not usually receive any professional care during their hospital stay [4,9,10]. Moreover, their quality of life during the hospital admission is low [8]. In addition, they may find it difficult to face the different situations associated with the illness and the dependence of their relative during hospitalization [11].

These reasons mean that hospitalization can be an experience full of stress and burnout for the family caregivers in the hospital, placing these caregivers in a very uncertain position [12,13]. Some studies focusing on these caregivers demonstrate that in this context, the stress of these caregivers may be higher than for those in the home environment [12]. All these circumstances can trigger negative emotional consequences in these caregivers [5,8,12,13,14,15].

This situation may be compounded when the dependence of the family member occurs suddenly, with the appearance of pathologies such as cerebrovascular accidents, cancer, or some forms of dementia, because the adaptation process for the caregiver—which has a profound impact regardless [16]—coincides with the hospital admission. These first moments of providing care are very stressful for the caregiver, as the needs they take on are multiple and complex [16,17,18]. As well as feeling required to make significant changes in their life, they take on the role of the caregiver with minimal preparation [5]. All this may be worsened with the sudden hospitalization of the patient and the initiation of caregiving in this environment, placing the caregiver in a position of increased vulnerability and stress caused by caregiving in an unknown environment [13]. 

The consequences of caring for a dependent older adult relative is a subject that has been widely addressed in the home environment [2,19,20,21], but not in the hospital environment [15], and much less with a focus on caregivers that debut in caregiving in this context. The conditions of family caregivers in the hospital and at home are not the same, and it is necessary to explore if the stressors work in the same way in the two settings. In this study, we focus on anxiety as it is one of the lesser studied negative emotional consequences and it has a high impact and importance [22]. The systematic reviews by Loh et al. [19] and Sallim et al. [20] reported a prevalence of anxiety of 21.4% in caregivers of relatives who have suffered a stroke [19], and 43.6% in caregivers of relatives who suffer from dementia [20]. To our knowledge, there are no studies that analyse the levels of anxiety and its determinants in family caregivers in the hospital. Knowing how anxiety works in family caregivers in the hospital may allow us to design interventions aimed at prevention or early detection of this problem in a collective that does not usually receive any attention.

### 1.2. Association between Burden and Anxiety

The appearance of stress in caregivers is associated with the stress caused by providing care. The majority of models and theories that aim to explain stress as a consequence of providing care for a relative are based on the Lazarus and Folkman transactional theory of stress [23]. According to these authors, the caregiving itself is not stressful; rather, it is the perception of the caregiving situation as being negative by the caregiver that triggers the appearance of stress and other negative emotional consequences [23,24].

In the informal care of dependent older persons, the model most used is the multi-dimensional stress process model by Pearlin et al. [25]. These authors maintain that the appearance and proliferation of stress are related, among others, with the primary objective stressors (i.e., the objective burden) and the primary subjective stressors (i.e., the subjective burden) [23].

The primary objective stressors, or objective burden, are constituted by the care needs of the care recipient (functional status, cognitive impairment, etc.) and behavioural problems, as well as the intensity of care provided by the caregiver. The primary subjective stressors, or subjective burden, are the perception of the caregiver of the demands of care as being stressful, resulting in the appearance of negative emotional consequences such as anxiety in these caregivers [25,26,27].

The subjective burden is defined as the state that is characterized by distress in several areas of the caregiver’s life, such as the caregiver’s health, psychological well-being, finances, social life, and the relationship between the caregiver and the care recipient resulting from the caregiving situation [28].

From the point of view of anxiety prevention, it is useful to know the variables that are associated with it in order to improve the health and quality of life of those caregivers that newly start caregiving for dependent older relatives in the hospital setting. Although various studies have addressed this in the context of the home environment [27,29,30], to date, no conclusions have been drawn with a sufficient level of evidence. Furthermore, as we commented previously, there are no studies analysing these issues in family caregivers in the hospital. The identification of the determinants of anxiety in these caregivers, especially those who newly start providing care in hospital, may help us to design specific interventions addressed to these caregivers.

### 1.3. The Mediating Role of Subjective Burden

At the theoretical level [25], anxiety has been associated with objective burden. The empirical findings of various authors have associated anxiety with the subjective burden and the objective burden [27,31]. Moreover, the subjective burden has been associated with the objective burden [32,33,34].

**Figure 1:**
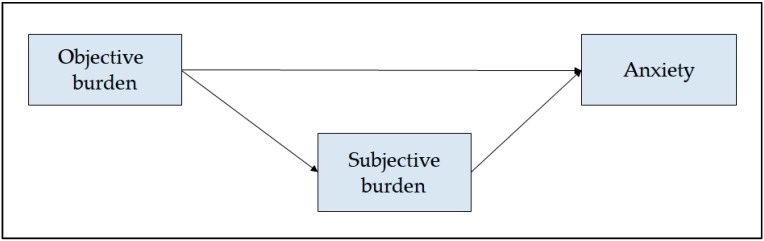
Conceptual model.

On the other hand, Kinght and Sayegh [35] proposed a common basic model within the care stress models, in which the subjective burden mediated the effects of the objective burden on the consequences derived from providing care, such as anxiety. These authors based their model on the following theoretical statements: (1) the consequences of stress are not only due to the direct effect of the objective burden [23], (2) these consequences are the result of the assessment of the situation by the caregiver [23,36], and (3) the subjective burden is directly related to anxiety [37]. Therefore, the subjective burden may buffer the effect of the objective burden on anxiety (Figure 1). Setting the status of these relationships may allow a better understanding of the caregiving process in general and particularly in family caregivers in the hospital.

Therefore, there is a need to evaluate and establish the relationship between objective burden, subjective burden and anxiety in caregivers of dependent older relatives that newly start providing care in hospitals, to better understand the process of care, and to identify the profiles of the caregivers at risk of developing anxiety; in this way, it would be possible to develop prevention and treatment programs to improve the health and quality of life of caregivers in this context.

With this study we aimed to: (1) determine the level of subjective burden and anxiety of caregivers of dependent older relatives, aged 65 years and older, that start providing care in the hospital, and (2) analyse the relationship between objective burden, subjective burden and anxiety in these caregivers. Specifically, we try to test the following hypotheses in caregivers of dependent older relatives that newly start providing care in hospital:

**Hypothesis 1** **(H1).** Objective burden (care recipient needs and intensity of care) is related to anxiety.

**Hypothesis 2** **(H2).** Subjective burden is related to anxiety.

**Hypothesis 3** **(H3).** Subjective burden mediates the relationship between objective burden and anxiety.

## 2. Materials and Methods

### 2.1. Design

This study was conducted using a descriptive cross-sectional design.

### 2.2. Participants

The study population was comprised of the main caregivers that started providing care for dependent older relatives admitted to the medium–long stay University Hospital in Jaen—Dr. Sagaz, Jaen (southern Spain). The inclusion criteria were: (1) have started providing care (been providing care for less than 2 months), (2) be providing care permanently, (3) not receiving any economic remuneration, (4) caring for a relative older than 65 years, and (5) the recipient of care is dependent in at least one of the ADLs. Participation in the study was offered to 72 caregivers that met the inclusion criteria, of which all 72 agreed to participate.

The sample size achieved gives a power of 97% to detect an r^2^ of at least 20% attributable to an independent variable in a linear regression that has been adjusted for 5 additional variables with an r^2^ of at least 1% with a statistical level of significance of 5%. This sample size meets the Nunnally and Berstein criteria for path analysis, which recommends a minimum of 10 participants per variable analysed [38].

### 2.3. Measurements

#### 2.3.1. Sociodemographic Variables

Caregiver: using an ad hoc questionnaire we collected data on age, sex, relationship (spouse, offspring, other), highest education level (without primary school, primary school, secondary school or university), employment (active, unemployed, retired, housewife, other), common residence with the recipient of care (yes, no).

Recipient of care: age, sex, principal diagnosis, reason for admission (cerebrovascular accident, cancer, cognitive deterioration, other).

#### 2.3.2. Dependent Variable

Caregiver anxiety was measured via the Hamilton anxiety scale [39]. This scale consists of 14 items. The interviewer scores each item from 0 to 4 points. The total score ranges between 0 and 56. The cut-off score proposed to detect clinical anxiety is 14 [40,41]. This scale was validated in Spain by Lobo et al. with good psychometric properties: internal consistency (Cronbach’s alpha of 0.89), test–re-test and interobserver reliability (intraclass correlation coefficient of 0.98 and 0.92, respectively) [42].

#### 2.3.3. Mediator Variable

Subjective burden of the caregiver was measured using the Robinson Caregiver Strain index [43]. This index consists of 13 questions with dichotomous responses (yes/no), with a total score between 0 and 13. This index has been validated in the Spanish population by López Alonso and Moral Serrano [44]. This index has good psychometric properties (Cronbach’s alpha of 0.86 and adequate construct validity) [44].

#### 2.3.4. Independent Variables

Objective burden was measured through the care recipient’s needs (functional capacity and cognitive impairment) and the intensity of care provided by caregivers measured by the number of basic ADLs assisted (ad hoc question) and hours of surveillance or supervision (ad hoc question). Functional capacity of the person cared for was measured with the Barthel Index [45]: a 10-item scale with a theoretic range from 0–100. This index was validated in Spain by Baztán et al. and has adequate psychometric properties (interobserver weighted kappa correlation coefficient of 0.98, and interobserver of 0.88) [46]. Cognitive impairment of the care recipient was evaluated with the Pfeiffer test and includes 10 dichotomous questions with a theoretical total score range of 0–10 [47]. This test was validated in Spain by Martínez de la Iglesia et al. and had a sensitivity of 85.7% and a specificity of 97.3% [48].

#### 2.3.5. Control Variable

Stressful life events: measured with the Holmes and Rahe Scale of Major Stressful Life Events [49]. This is a self-administered questionnaire that quantifies the life stressors that an individual has experienced. Twenty-four life events have been collated, and the person surveyed is asked to mark the events that have occurred in the last year. The test score is calculated by summing the points that correspond to each event.

### 2.4. Data Collection

Data were collected during the second semester of 2015 via a structured interview in order to ask the ad hoc questions related to the care provided (daily hours and number of ADLs, hours of surveillance and supervision, gender, relationship, and the sociodemographic data required to describe the study sample), and also to conduct the aforementioned questionnaires: Hamilton anxiety scale [39], Robinson Caregiver Strain index [43], Barthel Index [45], and Pfeiffer test [47]. The data were collected by highly qualified nurses, with at least five years of experience in the care of caregivers of dependent elderly. They were trained during a one five-hour session to guarantee the quality and uniformity of the data collection. This session included recommendations on the conducting of interviews, the use of the measurement instruments employed in the study, and the coding of data.

To guarantee the confidentiality of the information, all data collected in this project was recorded in an anonymous form, strictly following the current laws and regulations of data protection in Spain.

Additionally, informed consent was obtained from all participants in this study. Ethical approval for this study was obtained from the Institutional Review Board (0903201201).

### 2.5. Data Analysis

A descriptive analysis of the data was done to describe the sample. A bivariate analysis was performed and the linear Pearson correlation coefficient, or the Spearman correlation coefficient when the data did not meet the assumptions of normality, was calculated. For the multivariate analysis, path analysis was used which allowed us to include the effects of the mediator [50], and the simultaneous estimation of the associations between variables. To assess the model fit, the following were used: *p*-value, normalized chi square (X^2^/degree of freedom [df]), root mean square error of approximation (RMSEA), and its corresponding confidence interval, standardized root mean square residual (SRMR), and the comparative fit index (CFI). We selected these adjustment indices because they were found to be the least sensitive to sample size. For a good model fit, the above measures should be the following [51,52]: *p*-values above 0.10, X^2^/df values lower than 2, RMSEA values lower than 0.08, SRMR values lower than 0.05, and CFI values higher than 0.95. For the different statistical tests, the level of significance was set at 0.05. The analyses were performed using SPSS v. 22.0 (IBM Corp, Armonk, NY, USA) for descriptive analyses, transformation of the variables, and the bivariate analysis, and AMOS 18.0.0 (Amos development Corporation, Crawfordville, FL, USA) for Path analysis.

## 3. Results

### 3.1. Sample Population Description

In Table 1, we present the descriptive data of the sample characteristics and the variables used in this study. The profile of the care recipient was a person older than 75 years, male (63.9%), who was admitted to hospital for a cerebrovascular accident in 48.6% of cases. The dependence for basic ADLs had a mean score on the Barthel Index of 22. In terms of cognitive impairment, as measured by the Pfeiffer test, a mean score of 3.8 was obtained.

The profile of the family caregiver in the hospital that newly started in the care of dependent older persons aged 65 years and over was a mean age of 56 years, they were mostly women (79.2%), had not finished primary level studies (59.7%), 47.2% were the spouse of the care recipient, and 44.4% were offspring of the person cared for. In 65.3% of cases the caregiver lived with the recipient of care, and in 40.3% of cases the caregiver was a housewife.

In the hospital, these caregivers attended a mean of five basic ADLs and dedicated a mean of five hours to surveillance and/or supervision of the care recipient.

### 3.2. Levels of Burden and Anxiety

In terms of the subjective burden, as measured by the Robinson Caregiver Strain Index, a mean score of 5.4 was measured. Using a cut-off score of seven, 36.1% of the caregivers presented with subjective burden.

With respect to anxiety, as measured by the Hamilton anxiety questionnaire, a mean score of 15.4 was obtained. Using a cut-off score of 14, 14.86% of caregivers presented with anxiety.

### 3.3. Bivariate Analysis

The results of the bivariate analysis are shown in Table 2, where we can see that anxiety is positively correlated with subjective burden (r = 0.614, *p* = 0.001). Subjective burden also correlates positively with the number of basic ADLs attended (r = 0.397, *p* = 0.001), with the hours of surveillance or supervision in the hospital (r = 0.305, *p* = 0.001), and with the cognitive impairment in the care recipient (r = 0.510, *p* < 0.001).

### 3.4. Multivariate Analysis

**Figure 2:**
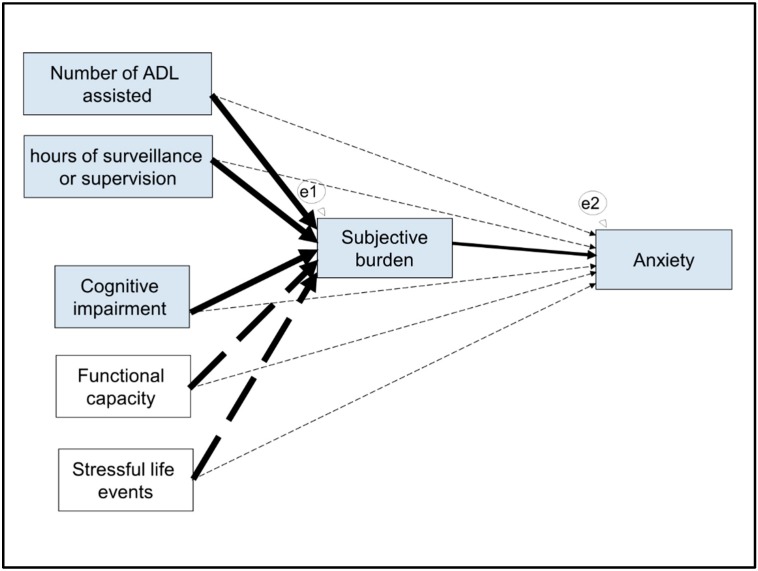
The starting model. Notes: e1 and e2 are latent errors. Arrows with a discontinuous line are non-significant pathways. Thin arrows correspond to relationships of hypothesis (1). Medium arrows correspond to relationships of hypothesis (2). Thick arrows correspond to relationships of hypothesis (3). Abbreviation: ADL, activities of daily living.

**Figure 3:**
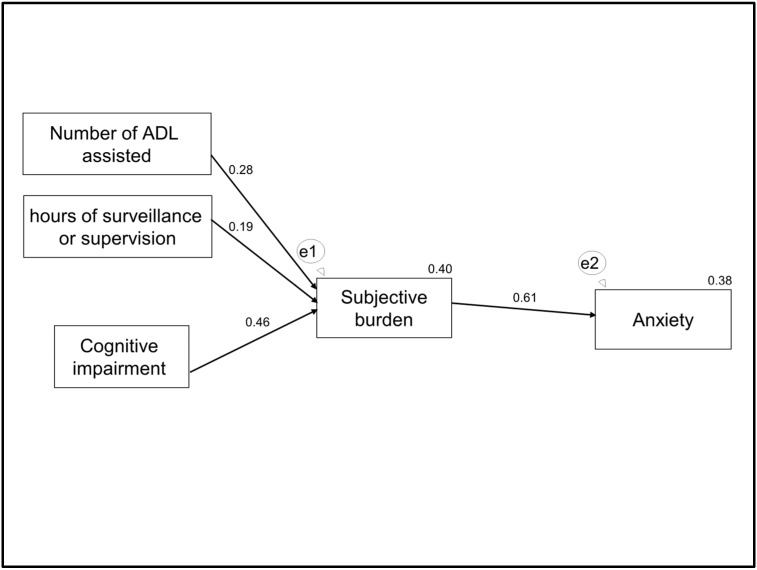
The final model. Notes: numbers by arrows represent standardised regression coefficients; numbers on the right upper corner of the boxes represent r^2^ of the partial regression of each variable. Notes: e1 and e2 are latent errors. Abbreviation: ADL, activities of daily living.

In terms of the path analysis, in Figure 2 we present the starting model, which was constructed based on the conceptual model explained in the introduction and which also includes the control variable—stressful life events. This module cannot produce goodness-of-fit indices as it has zero degrees of freedom. When we removed the non-significant pathways (arrows with a discontinuous line), we obtained the final model (Figure 3), with good fit indices (X² = 1.886; *p* = 0.596; df = 3; X²/df = 0.629; RMSEA = <0.001 (95% CI, <0.001–0.168; *p* = 0.662); SRMR = 0.031; CFI = 1.00).

The results obtained in the path analysis (Figure 3) show that the subjective burden was positively associated with the number of basic ADLs attended, the hours of surveillance or supervision by the caregiver, and the cognitive impairment of the care recipient. Anxiety was also positively associated with subjective burden. The subjective burden mediated the effects of the number of basic ADLs attended, the hours of surveillance or supervision by the caregiver, and the cognitive impairment of the care recipient on anxiety. The model explained 40% of the variance of subjective burden and 38% of the variance of anxiety.

In Table 3 we present the direct, indirect and total effects on anxiety of the variables in the final model. In this table we can see that the variables with the greatest effect on anxiety were subjective burden of the caregiver and the cognitive impairment of the care recipient.

## 4. Discussion

In the present study, the level of subjective burden and anxiety was assessed in family caregivers of dependent older adults aged 65 years and over, who had recently started caregiving in the hospital, and the factors associated with anxiety in these caregivers were analysed.

This study on the determinants of anxiety in family caregivers of dependent older adults was conducted in the hospital context. To our knowledge, this is the first study which focuses on those caregivers that debut in caregiving in this setting.

In the present investigation, the characteristics of the caregivers that start caregiving in the hospital are similar to those in a study conducted in Spain by the Institute for the Elderly and Social Services (IMSERSO, acronym of the Spanish name of the Institute), which investigated the care of elderly persons in their homes in Spain in 2005 [53]. Their study sample was representative at a national level. Therefore, the sample population in our study could be considered as representative of the caregivers of dependent older adults in Spain.

### 4.1. Levels of Burden and Anxiety

In the present study, the family caregivers presented with a level of burden of 36.1%, with a mean of 5.4 out of 13. Our results demonstrate a higher level of burden than that measured in other studies of hospital caregivers [12,15], but similar to studies of family caregivers in the home environment [26,33], with sociodemographic characteristics similar to the population sample in our study.

With regards to the level of anxiety, our results are similar to those presented in a systematic review by Sallim et al. on caregivers of patients suffering from Alzheimer disease [20], and are higher than those in a review by Loh et al. on caregivers of stroke survivors [19]. In the Spanish context, the level of anxiety found in our study is similar to that of studies conducted in the home environment [10,27].

Hence, family caregivers that have just started caregiving may have higher levels of burden and anxiety than those that have not recently started. Their level of burden also appears to be similar to those caregiving in the home environment.

Caregivers in the hospital could have theoretically less requirements than at home because several care recipient needs are attended by the healthcare team. However, for the caregiver, providing care in an unknown environment such as a hospital, even though in a lesser grade that at home, and being required to take on this care suddenly without information or previous training, may be more stressful than providing care in the home [5,12,13]. This possibly results in the appearance of negative emotional consequences in the caregiver, such as subjective burden or anxiety.

The levels of burden and anxiety found in our study highlight the importance of these problems and the need that debut caregivers in the hospital should be preferentially attended to in order to prevent these consequences.

### 4.2. Objective Burden, Burden, and Anxiety

In terms of the factors related to anxiety, after adjusting for possible confounding factors in the path analysis, we found that the number of basic ADLs attended, the hours of surveillance or supervision by the caregiver, the cognitive impairment of the recipient of care, and the subjective burden of the caregiver were related to higher levels of anxiety; and that the subjective burden mediated the effects of the number of basic ADLs attended, the hours of surveillance or supervision by the caregiver, and the cognitive impairment of the recipient of care on anxiety. Thus, hypotheses (2) and (3) have been demonstrated. Regarding hypothesis (1), only indirect relationships via subjective burden have been demonstrated between objective burden and anxiety. All the primary objective stressors were related with anxiety except functional capacity.

The findings in this study highlight a positive direct effect of subjective burden on anxiety, after adjusting for objective burden, and are consistent with other studies focused on caregivers in the home (both cross-sectional [10,27,54] as well as longitudinal [55]) that had valid and reliable measurements that were adjusted for objective burden. Hence, our study increases the available evidence of the possible harmful effect of subjective burden on anxiety, expanding this result to family caregivers in the hospital that newly start to provide care and controlling for stressful life events.

On the other hand, the results of this study demonstrate that the objective burden has a positive effect on anxiety, which is mediated by the subjective burden. This result is consistent with the conclusions of other studies conducted with caregivers in the home setting [27,31]. This investigation increases the evidence that the subjective burden can buffer the effects of the objective burden on anxiety, expanding these findings to family caregivers that start providing care in the hospital. This finding is consistent with the Lazarus and Folkman stress theory [23], which explains that the effect of stressors on emotional health depends on the perception of the person of these stressors and to what extent these stressors are identified as threats or being insurmountable. Given that subjective burden is the result of the perception of the stressors in the care providing situation [36], this burden can cushion or augment the effect of the objective burden on anxiety depending on whether this perception is positive (low burden) or negative (high burden). In our study, a greater intensity of care for relatives with cognitive impairment was accompanied by a higher burden and increased anxiety.

The direct proportional relationship between the objective burden and the subjective burden has been demonstrated with a sufficient level of evidence by various systematic reviews conducted on studies of caregivers in the home [34,37], as well as two studies conducted in the hospital with family caregivers [5,15]. Therefore, our study increases the evidence available on the relationship between the objective burden and the subjective burden in caregivers of dependent older adult relatives in the hospital setting.

Thus, family caregivers that start providing care in the hospital may experience stressing situations derived from the attention given to their relative and face these situations in a similar way to those at home, so that the greater attention and responsibility in caregiving is related to greater subjective burden and anxiety, and the perception and assessment of the caregiving situation may buffer the relation between this situation and anxiety.

In general, studies that analyse the experiences of family caregivers in the hospital are scarce [15]. Our results support the fact that family caregivers that start providing care in the hospital should be treated as recipients of care by healthcare professionals. Informal care in the hospital should be carried out on a voluntary basis—never in the perspective of being assigned to the relatives’ specific and systematized tasks. The goal is to provide better comfort and well-being to the recipient of care and, on the other hand, to reduce family distress levels. When present at the hospital, the care plan should also include specific interventions addressed to caregivers, especially if they are starting as caregivers of older adult relatives. Due to the possible buffering effect of subjective burden, it could be appropriate to screen this subjective burden in family caregivers of older adult relatives that start providing care in the hospital.

This study has two limitations. The first is due to its cross-sectional nature, which means that it is not possible to establish a causal relationship between the variables studied. Nevertheless, this limitation is somewhat attenuated by using path analysis, which allows the establishment of hypotheses regarding the causal pathways present in the study and the direction of the effect between the variables studied. The second limitation is due to the population sample used, which was a convenience sample. This could result in problems of representativeness of our sample. However, the similarity between our study population and those of samples representative at a national level and the absence of participants who declined participation in the initial sample means that we can consider our study sample to be sufficiently representative.

## 5. Conclusions

Despite the limitations mentioned, we can conclude that in family caregivers of dependent older adult relatives that debut caregiving in hospital:

- The levels of subjective burden and anxiety may be higher than in those that have not recently started caregiving, highlighting the importance of these problems and the need that debut caregivers in the hospital should be preferentially attended to in order to prevent these consequences.
- Subjective burden is a possible risk factor for anxiety, independent of the objective burden.
- Subjective burden may buffer the effects of objective burden on anxiety.

Therefore, debuting family caregivers of older adult relatives in the hospital should be treated as recipients of care by the healthcare professionals, as it is probably necessary to include specific interventions addressed to caregivers in the care plan in order to reduce distress levels and increase well-being. In this intervention, the screening of subjective burden may also be useful to anxiety prevention.

## Figures and Tables

**Table 1:** Descriptive data of the sample characteristics and the variables used in this study.

	Mean (SD)	Theoretical Range	Practical Range	*n* (%)	95% CI
**Care recipient**					
Age	75.5 (8.2)		60–92		73.27–77.73
Gender					
Male				46 (63.9%)	52.10–75.67
Female				26 (36.1%)	24.32–47.90
Main pathology					
Stroke				35 (48.6%)	36.37–60.85
Cancer				15 (20.8%)	10.75–30.90
Dementia				1 (1.4%)	0.03–7.49
Other				21 (29.2%)	17.97–40.36
Functional capacity	22 (20.9)	0–100	0–90		16.27–27.66
Cognitive impairment	3.8 (3.1)	0–10	0–10		4.00–4.71
**Caregiver**					
Age	56 (14.7)		23–4		52.57–59.49
Gender					
Female				57 (79.2%)	69.09–89.24
Male				15 (20.8%)	10.75–30.90
Relationship					
Spouse				34 (47.2%)	34.99–59.44
Offspring				32 (44.4%)	32.27–56.61
Other				6 (8.3%)	1.25–15.44
Education level					
No primary school				43 (59.7%)	47.69–71.74
Secondary school				21 (29.2%)	17.93–40.36
University				8 (11.1%)	3.15–19.06
Employment status					
Active				20 (27.8%)	16.73–38.81
Unemployed				12 (16.7%)	7.36–25.696
Retired				10 (13.9%)	5.20–22.57
Housewife				29 (40.3%)	28.25–52.30
Other				1 (1.4%)	0.03–7.49
Common residence				47 (65.3%)	53.58–76.96
Number of ADLs assisted	4.8 (3.1)		1–10		4.06–5.52
Hours of surveillance or supervision	5.5 (4.9)		1–10		4.37–6.69
Stressful life events	96.2 (47.3)		44–239		85.11–107.36
Anxiety	15.4 (10)	0–56	0–46		13.03–17.72
Subjective burden	5.4 (3.1)	0–13	0–12		4.67–6.11

Abbreviations: SD, standard deviation; CI, confidence interval; ADL, activities of daily living.

**Table 2:** Correlation matrix and descriptive data of the variables used in the models.

	2	3	4	5	6	7
1. Anxiety	0.614 **	0.212	0.225	0.012	−0.135	0.232
2. Subjective burden		0.397 **	0.305 **	0.010	−0.220	0.510 **
3. Number of ADL assisted			0.433 **	0.114	−0.088	0.121
4. Hours of surveillance or supervision				0.079	0.222	0.080
5. Stressful life event					0.032	−0.196
6. Functional capacity						−0.452 **
7. Cognitive impairment						

** The correlation is significant at level 0.01 (bilateral); Abbreviations: ADL, activities of daily living.

**Table 3:** Standardized direct, indirect, and total effects of variables on anxiety in the final model.

	Direct Effects	Indirect Effects	Total Effect
Subjective burden	0.61	0	0.61
Number of ADL assisted	0	0.17	0.17
Hours of surveillance or supervision	0	0.11	0.11
Cognitive impairment	0	0.24	0.24

Abbreviation: ADL, activities of daily living.
